# Knowledge of Risk Factors, Symptoms, Treatment, and Prevention of Postoperative Urinary Retention Among the General Population of the Western Region of Saudi Arabia

**DOI:** 10.7759/cureus.89129

**Published:** 2025-07-31

**Authors:** Amani O Safdar, Atheer M Aljuaid, Waad I Barnawi, Thekra A Alwafi, Zeyad O Alsehemi

**Affiliations:** 1 Medicine, Umm Al-Qura University, Makkah, SAU; 2 Surgery, Medicine, Umm Al-Qura University, Makkah, SAU

**Keywords:** kingdom of saudi arabia (ksa), pain control, postoperative complications, postoperative urinary retention, prostate

## Abstract

Background: After an operation, patients may experience a variety of complications, including postoperative urinary retention (POUR). POUR is a frequent complication that patients experience after surgical interventions and is defined as the inability to voluntarily void despite having a filled bladder. This study evaluated the awareness of POUR among the citizens of the western region of Saudi Arabia.

Methods: A descriptive cross-sectional study was done in the western region of Saudi Arabia from October 2024 to January 2025. A total of 500 adults who are living in the region were included in this study. Sociodemographic factors, surgical history, and knowledge of POUR were evaluated using chi-squared tests, mean and standard deviation analysis, and Spearman’s correlation test.

Results: A majority of participants (49.8%) were aware of the term POUR, and 74.5% of them correctly defined POUR as the inability to urinate following a surgical procedure despite having a full bladder or the urge to urinate. Furthermore, most of the participants addressed the symptoms of POUR as the urge to urinate accompanied by an inability to void (85.9%), painful inability to void (56.6%), and suprapubic or bladder pain or discomfort (36.7%). Reported risk factors included renal disorders (37.4%), diabetes (23.2%), neurological disorders (15.7%), and hypertension (14.4%).

Conclusion: This study highlights that there is moderate awareness of POUR in the region, with a lack of awareness of its symptoms, risk factors, and prevention strategies. Early identification and education are essential to prevent further complications.

## Introduction

Postoperative urinary retention (POUR) is a common and often serious complication that can occur after surgical interventions and is characterized by the inability to voluntarily void urine despite having a filled bladder [1]. In the western region of Saudi Arabia, the healthcare sector is rapidly evolving to accommodate increasing surgical demands. Thus, to optimize patient outcomes, it is important to have a deep understanding of POUR, including its risk factors, clinical manifestations, therapeutic interventions, and prevention strategies.

Epidemiological studies showed that the prevalence of POUR exhibits a wide variability, with reported rates ranging from 5% to 70% [2,3]. This wide variety of rates depends on many factors, such as the type and duration of the surgical procedure, the type of anesthesia, the medications used in the perioperative period, and patient-specific aspects (e.g., sex and underlying comorbidities) [3-5]. Males have a higher risk following procedures involving the prostate, which is attributable to anatomical and physiological differences [3]. Comorbidities such as diabetes, obesity, and neurological disorders can also increase the risk of having POUR [1].

The type of anesthesia, whether it is general or regional, can significantly contribute to the incidence of POUR. During general anesthesia, it is often necessary to use muscle relaxants, which can compromise bladder tone and contractility, thereby increasing the risk of POUR. Similarly, regional anesthesia techniques, such as spinal or epidural anesthesia, are useful in many surgical procedures but may disrupt the sensory and motor pathways that control bladder function [6]. Thus, an appropriate anesthetic technique must be chosen while balancing effective perioperative pain management with the potential for urinary complications.

Delays in recognition of POUR and its related symptoms can lead to significant morbidity and prolong the length of hospital stay [7,8]. The diagnostic approach is commonly done by measuring the post-void residual urine by bladder ultrasound, along with detailed patient history and physical examination to detect risk factors [3].

To date, no research has assessed the public knowledge and awareness of POUR in the western region of Saudi Arabia. It is important to educate the population about POUR, as this complication can have a major impact on patient recovery and utilization of healthcare resources. Furthermore, providing patients and their caregivers with comprehensive information can facilitate early recognition and prompt reporting of issues, as well as enhance collaboration with healthcare providers, which will ultimately help to optimize the recovery process [3].

From the perspective of the healthcare system, improved public knowledge may also lead to more efficient resource allocation, reduced lengths of hospital stay, and lower overall costs associated with the management of this common postoperative complication [2]. Therefore, serious efforts are needed to disseminate evidence-based educational materials and promote open dialogue between patients, their families, and medical professionals. Such efforts are essential to address the burden of POUR and elevate the standard of surgical care within the western region of Saudi Arabia. Thus, the aim of this study is to provide a comprehensive understanding of the risk factors, symptoms, treatment options, and preventive strategies for POUR among the general population of the western region of Saudi Arabia.

## Materials and methods

Methods

This descriptive cross-sectional study was performed in the western region of Saudi Arabia from October 2024 to January 2025. Participants were included if they were adults aged 18 years and older of both genders who were residents of the western region, including areas such as Makkah, Jeddah, Taif, and Madinah; able to read and understand either Arabic or English; provided informed consent; and completed the entire questionnaire. Individuals were excluded if they were under 18 years of age, resided outside the western region of Saudi Arabia, were unable to understand Arabic or English, did not provide consent, or submitted incomplete responses.

The questionnaire was randomly distributed via social media platforms. Using OpenEpi (https://www.openepi.com/), the appropriate sample size was calculated as 385 participants with 5% margin of error and 95% confidence interval [9]. In case of possible data loss, the minimum sample size was increased to 500 respondents.

The questionnaire was created as an online survey in Arabic. It was developed using Google Forms and sent electronically to the participants. The questionnaire contained the objectives of the study, information about the target population, and a form for consent to participate. The consent form was filled out by all participants before starting the questionnaire.

The questionnaire consisted of four parts. The first part concerned sociodemographic data, such as age, gender, nationality, and educational level, as well as work in the medical field and health education about surgical complications. The second part asked a series of questions about the respondents' surgical history. The third part contained questions to evaluate the knowledge of the respondents about POUR. The last part included questions that assessed the respondents' knowledge regarding its symptoms. The data were collected electronically and did not include any personal data, such as names, email addresses, or phone numbers. The researchers received all responses anonymously.

Once all responses were collected, they were automatically transferred into an Excel sheet. The data were analyzed using Statistical Product and Service Solutions (SPSS, version 26; IBM SPSS Statistics for Windows, Armonk, NY). To assess the relation among the variables, the chi-squared test was applied for qualitative data that were presented as numbers and percentages. The mean and standard deviation (SD) were used for quantitative variables. Correlation analysis was carried out using Spearman's test. A p-value of less than 0.05 was considered statistically significant.

## Results

Of the 548 participants, 73.2% were 18-30 years old, 61.9% were females, 96.5% had Saudi nationality, and 76.5% had a university level of education. Furthermore, 35.2% were working in the medical field, and 31.9% had previous health education about surgical complications, as shown in Table 1. Table 2 shows that 190 (34.7%) of the participants had a previous surgical procedure. Of those, 7.3% had a hernial repair, 6.8% had an appendectomy, and 6.3% had gallbladder surgery. For 35.3%, the duration of surgery was 30-60 minutes, 30% used a Foley catheter, and 76.4% had general anesthesia. The majority (73.7%) had self-reported POUR as they experienced inability to urinate after surgery despite having a full bladder or desire to urinate, and for 89.4% of them, the retention lasted for a few hours.

**Table 1:** Distribution of the studied participants according to their demographic characteristic, working in the medical field and having health education about surgical complications No. 548

Variable	No. (%)
Age (years)	
18-30	401 (73.2)
31-40	46 (8.4)
41-50	50 (9.1)
>50	51 (9.3)
Gender	
Female	339 (61.9)
Male	209 (38.1)
Nationality	
Saudi	529 (96.5)
Non-Saudi	19 (3.5)
Educational Level	
Secondary School or Less	98 (17.9)
University	419 (76.5)
Postgraduate	31 (5.7)
Working in the medical field?	
No	355 (64.8)
Yes	193 (35.2)
Had health education about surgical complications?	
No	373 (68.1)
Yes	175 (31.9)

**Table 2:** Distribution of the studied participants according to surgical history No. 548

Variable	No. (%)
Surgical History	
Have you ever had surgery/ surgical procedure?	
No	358 (65.3)
Yes	190 (34.7)
For those who had a previous surgery/ surgical procedure (No. 190)	
Types of Surgery	
Bariatric Surgery	6 (3.1)
Hernia Repair	14 (7.3)
Appendectomy	13 (6.8)
Breast Surgery	3 (1.5)
Colorectal Surgery	3 (1.5)
Thyroid Surgery	1 (0.5)
Gallbladder Surgery	12 (6.3)
Other	138 (73.2)
Duration of Surgery	
<30 min	46 (24.2)
30-60 min	68 (35.9)
60-120 min	38 (20)
120-180 min	15 (7.8)
>180 min	23 (12.1)
Use of a Foley Catheter	
No	133 (70)
Yes	57 (30)
Type of Anastasia	
General	142 (76.4)
Local	27 (14.2)
Epidural	18 (9.4)
Have you ever had postoperative urinary retention?	
No	50 (26.3)
Yes	140 (73.7)
If yes, specify duration of post-operative urinary retention (No. 140)	
Few hours	125 (89.4)
1 day	12 (8.5)
> 1 day	3 (2.1)

Table 3 shows the participants’ responses to knowledge items related to POUR. It was found that 49.8% had heard of POUR, and 74.5% correctly identified it as the inability to urinate after surgery, despite having a full bladder or desire to urinate. The most known symptoms were the need to urinate with an inability to void (85.9%), painful inability to void (56.6%), and suprapubic or bladder pain or discomfort (36.7%).

**Table 3:** Participants’ response to knowledge items about postoperative urinary retention No. 548

Variable	No. (%)
Have you ever heard of postoperative urinary retention?	
No	275 (50.2)
Yes	273 (49.8)
What do you think is the meaning of postoperative urinary retention?	
Inability to urinate after surgery despite having full bladder or desire to urinate *	408 (74.5)
Loss of bladder control after surgery	24 (4.4)
Abnormal increase in frequency of urination after surgery	1 (0.2)
Urination only with small amounts after surgery	20 (2.6)
Loss of desire to urinate after surgery	6 (1.1)
I don’t know	89 (16.2)
What is considered as a postoperative urinary retention symptom?	
Painful inability to void *	310 (56.6)
Need to urinate with inability to void *	471 (85.9)
Restless and distress *	229 (41.8)
Suprapubic or bladder pain/discomfort *	201 (36.7)
Palpable bladder (swelling) *	117 (21.4)
Passing small amounts of urine with blood in urine	83 (15.1)
Fever	49 (8.9)
Flanks pain	100 (18.2)
I don’t know	123 (22.4)
What are causes of postoperative urinary retention?	
Injury to any of (kidneys, ureters, bladder, urethra) during operation *	123 (22.4)
Nerves injury during the operation *	134 (24.5)
Spinal cord injury during the operation *	47 (8.6)
Spinal cord compression *	39 (7.1)
Medical mistake by Anastasia (over dose, wrong drug, wrong technique) *	81 (14.8)
Common Side effects of Anastasia*	214 (39.1)
Inflammation*	87 (15.9)
No specific cause	45 (8.2)
I don’t know	152 (27.7)
What are risk factors of postoperative urinary retention?	
Age *	189 (34.5)
Male *	58 (10.6)
Female	38 (6.9)
Familial *	21 (3.8)
Renal disease (stones, infections, failure, …) *	205 (37.4)
DM *	127 (23.2)
HTN *	79 (14.4)
Neurological disorders *	86 (15.7)
Longer surgery *	123 (22.4)
Multiple surgeries *	64 (11.7)
Previous postoperative urinary retention *	147 (26.8)
General Anastasia *	124 (22.6)
Spinal Anastasia *	69 (12.6)
I don’t know	187 (34.1)
What are methods of postoperative urinary retention prevention?	
Ambulation (walking) *	207 (37.8)
Reduce fluid *	143 (26.1)
Incentive spirometry *	33 (6)
Prophylactic medication *	159 (29)
Can not prevent it	37 (6.8)
I don’t know	225 (41.1)
What are methods of postoperative urinary retention treatment?	
Catheterization *	220 (40.1)
Medication *	156 (28.5)
Hot therapy (warm compress) *	95 (17.3)
Surgery	36 (6.6)
Only monitoring, self-resolving	141 (25.7)
No treatment available	20 (3.6)
I don’t know	183 (33.4)
What are complications of postoperative urinary retention?	
Bladder perforation *	137 (25)
UTI *	288 (52.6)
Renal failure *	185 (33.8)
Incontinence *	84 (15.3)
Renal stone	88 (16.1)
I don’t know	185 (33.8)

Regarding the causes of POUR, 39.1% reported that it is a common side effect of anesthesia, 24.5% reported nerve injury during the operation, and 22.4% reported that it is caused by an injury to the kidneys, ureters, bladder, or urethra during the operation. Regarding risk factors, 37.4% reported renal disease (stones, infections, failure, etc.), 34.5% reported age, and 26.8% reported previous POUR. About 37.8% reported that ambulation (walking) is a method of prevention, while 29% mentioned the use of prophylactic medications. Most of them (40.1%) reported catheterization as a method of treatment, followed by medications (28.5%). The most commonly known POUR complications were urinary tract infection (52.6%) and renal failure (33.8%).

**Figure 1:**
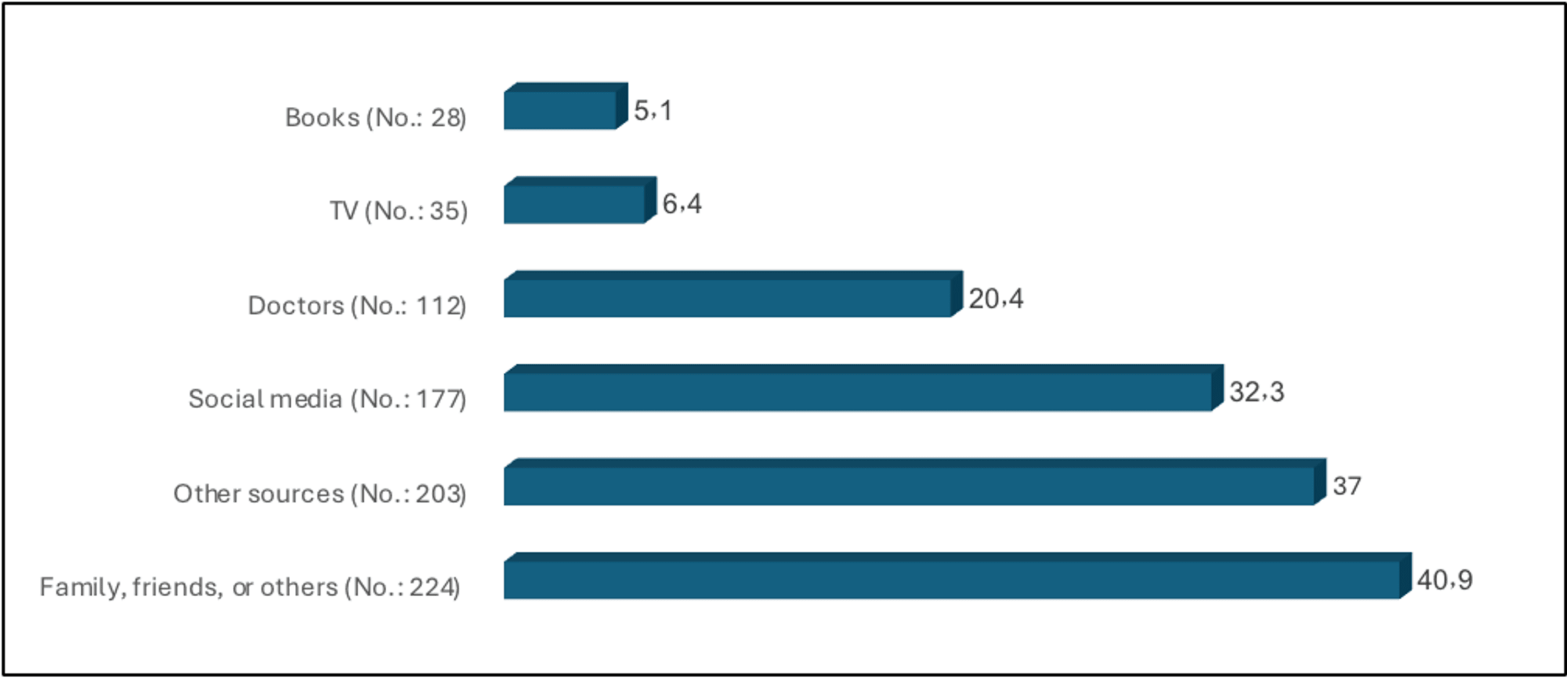
Sources of knowledge about postoperative urinary retention No. 548

**Figure 2:**
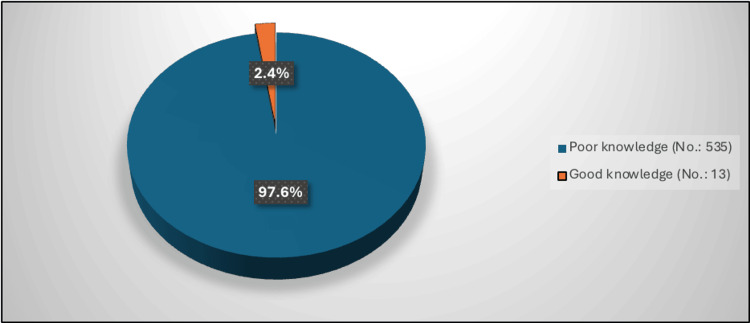
Percentage distribution of the level of knowledge about postoperative urinary retention No. 548

The most common sources of knowledge about POUR among the participants were family, friends, or others (40.9%), social media (32.3%), and doctors (20.4%) (Figure 1). The mean knowledge score was 10.58 ± 5.49. Figure 2 demonstrates that only 2.4% of the participants had a good level of knowledge about POUR with a score of 23 or higher out of 38, while the majority (97.6%) had poor knowledge.

**Figure 3:**
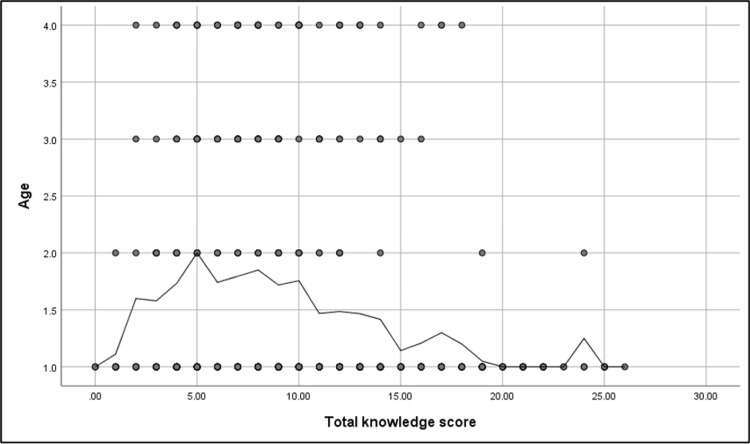
Spearman’s correlation analysis between the total knowledge score and participants’ age N.B.: (r = -0.24; p ≤ 0.001)

Table 4 shows that the prevalence of good knowledge about POUR was significantly higher among participants working in the medical field (69.2%) and those who had previous health education about surgical complications (76.9%) (p ≤ 0.05). A non-significant relationship was found between the knowledge level and participants’ demographics (p ≥ 0.05). Figure 3 shows that there was a significant negative correlation between the total knowledge score and participants’ age (r = -0.24; p ≤ 0.001). Table 5 illustrates that the prevalence of good knowledge was significantly higher among participants who had a previous surgery with a duration of POUR of a few hours (p ≤ 0.05).

**Table 4:** Relationship between level of knowledge about postoperative urinary retention and participants; demographics, working in the medical field and having health education about surgical complications No. 548

Variable	Knowledge level		χ^2^	p-value
	No. (%)	No. (%)		
Age (years)				
18-30	389 (72.7)	12 (92.3)	3.12	0.372
31-40	45 (8.4)	1 (7.7)		
41-50	50 (9.3)	0 (0.0)		
>50	51 (9.5)	0 (0.0)		
Gender				
Female	331 (61.9)	8 (61.5)	0.01	0.981
Male	304 (38.1)	5 (38.5)		
Nationality				
Saudi	516 (96.4)	13 (100)	0.47	0.489
Non-Saudi	19 (3.6)	0 (0.0)		
Educational level				
Secondary school or less	95 (17.8)	3 (23.1)	0.38	0.823
University	410 (76.6)	9 (96.2)		
Postgraduate	30 (5.6)	1 (7.7)		
Working in the medical field?				
No	351 (65.6)	4 (30.8)	6.75	0.009
Yes	184 (34.4)	9 (69.2)		
Had health education about surgical complications?				
No	370 (79.2)	3 (23.1)	12.39	<0.001
Yes	165 (30.8)	10 (76.9)		

**Table 5:** Relationship between the level of knowledge about postoperative urinary retention and surgical history No. 548

Variable	Knowledge level		χ^2^	p-value
	No. (%)	No. (%)		
Surgical History				
Have you ever had surgery/ surgical procedure?				
No	349 (65.2)	9 (69.2)	0.09	0.765
Yes	186 (34.8)	4 (30.8)		
For those who had previous surgery/ surgical procedure (No. 190)				
Types of Surgery				
Bariatric Surgery	6 (1.1)	0 (0.0)	2.86	0.492
Hernia Repair	14 (2.6)	0 (0.0)		
Appendectomy	13 (2.4)	0 (0.0)		
Breast Surgery	3 (0.6)	0 (0.0)		
Colorectal Surgery	3 (0.6)	0 (0.0)		
Thyroid Surgery	1 (0.2)	0 (0.0)		
Gallbladder Surgery	11 (2.1)	1 (7.7)		
Other	135 (25.2)	3 (23.1)		
Duration of Surgery				
<30 min	45 (8.4)	1 (7.7)	5.53	0.354
30-60 min	67 (12.5)	1 (7.7)		
60-120 min	38 (7.1)	0 (0.0)		
120-180 min	15 (2.8)	0 (0.0)		
>180 min	21 (3.9)	2 (15.4)		
Use of a Foley Catheter				
No	131 (24.5)	2 (15.4)	0.78	0.676
Yes	55 (10.3)	2 (15.4)		
Type of Anastasia				
General	139 (26)	3 (23.1)	0.78	0.94
Local	26 (4.9)	1 (7.7)		
Epidural	18 (3.4)	0 (0.0)		
Have you ever had postoperative urinary retention?				
No	49 (9.2)	1 (7.7)	0.09	0.955
Yes	137 (25.6)	3 (23.1)		
If yes, specify duration of post-operative urinary retention (No. 140)				
Few hours	123 (23)	2 (15.4)	13.04	0.005
1 day	12 (2.2)	0 (0.0)		
> 1 day	2 (0.4)	1 (7.7)		

## Discussion

The purpose of this study was to evaluate the awareness of POUR among the citizens of the western region of Saudi Arabia. A descriptive cross-sectional study was done for this purpose. The main results showed an acceptable level of knowledge regarding POUR within the study participants. Our findings highlight a critical knowledge gap, with only 2.4% of participants demonstrating a good level of knowledge about POUR. Although 74.5% of participants correctly identified its definition and recognized some key symptoms, the understanding of its risk factors, causes, and prevention methods was poor. This may affect patients’ ability to recognize complications early and seek medical help, potentially increasing the risk of urinary tract infections (UTIs), renal impairment, or extended hospitalization, which are complications well documented in previous studies [2,6,8].

Surprisingly, a significant number of participants (73.7%) experienced POUR as a postoperative complication, with 89.4% of these cases persisting for a few hours. Previous research has reported much lower rates. For example, a study conducted in 2024 in the Republic of Moldova indicated that the prevalence of POUR ranged from 5.5% to 7.9% among the study participants [10]. Another study conducted in Turkey in 2020 revealed that 9.9% of the population had POUR [11]. Additionally, 4.2% of participants in a study conducted at Mary’s Hospital in London experienced POUR in 2018 [12]. The significant discrepancies in reported percentages of POUR may have resulted from variations in demographics, comorbidity, surgery type and duration, anesthesia methods, and both preoperative and postoperative care, among other factors. Nevertheless, because of the self-reported nature of the study, there is a possibility that participants may have misunderstood or misidentified POUR, potentially confusing it with other urinary symptoms, which may have affected the accuracy of the reported prevalence. This necessitates further analysis and research to clarify the underlying factors contributing to this variation. Due to these discrepancies, further research is also necessary to assess the nationwide prevalence and investigate the underlying reasons for the elevated results.

Our results also show that, while 74.5% of participants correctly defined POUR, awareness of its causes was inadequate. Only 39.1% recognized anesthesia as a possible cause. A figure consistent with findings from Baldini et al. [6], who demonstrate the role of general and regional anesthesia in affecting bladder function. Moreover, despite evidence showing that male gender, older age, and comorbid conditions such as diabetes and neurologic disorders increase the risk of POUR [1,3,4], these were poorly recognized by participants in our study. For instance, only 10.6% and 15.7% of the participants identified male gender and neurologic disorders as risk factors, respectively. This highlights a significant knowledge gap in the community regarding important risk factors of POUR.

Approximately 37.8% reported that early ambulation may prevent POUR. This aligns with a previously published review that emphasizes early mobilization to stimulate normal bladder function [3]. However, 29% of the participants stated that medications might be helpful in preventing POUR, which implies that these participants may misunderstand the role of pharmacological treatments, as they are typically more effective for managing POUR rather than serving as a preventive measure.

The study also demonstrated that individuals working in the medical field and those who already have received health education about surgical complications were significantly more knowledgeable about POUR. This supports existing literature suggesting that targeted educational interventions, especially among high-risk populations, can substantially improve awareness and outcomes [13].

Our findings have important implications for both patient outcomes and healthcare systems. Improving public knowledge of POUR may enhance early symptom recognition, reduce the time to presentation, and therefore lower the incidence of complications. This may decrease hospital stays and the need for emergency interventions, improving resource utilization and overall surgical care efficiency. In addition, our results underscore the need for preoperative counseling that addresses common but less recognized complications such as POUR, especially for high-risk individuals.

This study has several limitations that warrant consideration. This study was limited to the western region of the Saudi Arabian population who speak Arabic or English. In addition, a majority of participants (73.2%) were between 18 and 30 years old, and 61.9% were females, indicating a potential sampling bias.

Finally, investigating the knowledge of POUR across all regions of Saudi Arabia is recommended to evaluate the necessity to enhance awareness and understanding of POUR among the general population for future prevention efforts.

## Conclusions

This study evaluated the awareness of POUR among the general population of the western region in Saudi Arabia. On the other hand, while awareness exists showing moderate knowledge levels of POUR and its symptoms, in-depth understanding of POUR remains low regarding knowledge about POUR’s risk factors and prevention measures. Therefore, it is recommended to conduct further studies among all regions of Saudi Arabia to assess the current knowledge of POUR and evaluate the need for raising awareness and knowledge to promote prevention and improve management strategies.

## Appendices

The Questionnaire

Please note that your participation is voluntary and all the data in this questionnaire is confidential and will only be used for research purposes.

Do you agree to participate? - Yes - No

1 - Demographic data

Age • Less than 18 years old • 18-30 years • 31-40 years • 41-50 years • 50 years or more Sex • Female • Male Nationality • Saudi • Non-Saudi

The level of education • High school and less • Bachelor's • Master's or PhD

 
Do you work in the field of healthcare? • Yes • No Have you got healthy education about the complications of surgeries? • Yes • No 2 - Surgical history Have you had surgery before? • Yes • No The type of surgery? • Appendectomy • Breast surgery • Cholecystectomy • Surgery in the colon and rectum • Thyroid gland surgery

• Bariatric surgery • Hernia repair surgery • Another The period of the surgery? • <30 minutes • 30-60 minutes • 60-120 minutes • 120-180 minutes • >180 minutes

Was the urinary catheter used? • No • Yes What kind of anesthesia is used during the process? (If you answer the previous question, yes) • General anesthesia • Local anesthesia • Spinal anesthesia • Epidural anesthesia

•Other Have you suffered from urine retention after surgery before? • Yes • No Duration of urine retention after surgery • A few hours • One day • > One day

3 - Evaluation of knowledge of urine retention after surgery Have you ever heard of urinary retention after surgery? • Yes • No What is your source of knowledge about urine retention after surgery? • Social media • Books • I heard about it (family, friends, others) • TV • Campaign • Doctors • Other What is the meaning of urinary retention after surgery? • Inability to urinate after surgery despite the full bladder or desire to urinate • Loss of control over bladder after surgery • An abnormal increase in the urination rate after surgery • Urinating in small quantities only after surgery • Loss of desire to urinate after surgery • Renal failure after surgery • I don't know

4 - Evaluation of knowledge of the symptoms of urine retention after surgery What are the symptoms that indicate urine retention after surgery? (Multiple answers can be chosen) • Inability to urinate due to pain • The need to urinate with inability to urinate • Pain/discomfort above the pubic or bladder •Swelling of the bladder is tangible • Discomfort and distress • Pain in the loin • Fever • The passage of small amounts of blood with urine • I don't know In your view, what are the reasons for urine retention after surgery? (Multiple answers can be chosen) ▪ Any (kidney, ureter, bladder, urethra) injury during the operation ▪ Nerve injury during the operation ▪ Spinal cord injury during the operation ▪ Pressure on the spinal cord ▪ Medical error by anesthesia (an overdose, wrong drug, wrong technique) ▪ Common side effects of anesthesia • Other medicines • Inflammation ▪ There is no specific reason ▪ I don't know What are the risk factors that cause urine retention after surgery? (Multiple answers can be chosen) • Age ▪ Male gender ▪ Female gender •Genetic ▪ Kidney disease (stones, infection, failure, ...) •Diabetes ▪ High blood pressure ▪ Neurological disorders ▪ The duration of the surgery ▪ Multiple surgeries ▪ Previous diuretics after surgery. • General anesthesia ▪ Spinal anesthesia ▪ I don't know In your opinion, what are the ways to prevent urine retention after surgery? (Multiple answers can be chosen) • Walking and mobilization ▪ incentives spirometry ▪ Reducing fluids ▪ Preventive drugs ▪ It can not be prevented ▪ I don't know In your opinion, what are the ways to treat urine retention after surgery? (Multiple answers can be chosen) ▪ Catheterization ▪ Medication ▪ Warm compresses • Surgery ▪ The patient remains under observation only, it is automatically improving ▪ There is no treatment available ▪ I don't know In your opinion, what are the complications of urine retention after surgery? (Multiple answers can be chosen) ▪ Urinary bladder perforation ▪ Renal failure • Urinary tract inflammation ▪ Urinary incontinence • Kidney stones ▪ I don't know
